# An Improved ResNet-1d with Channel Attention for Tool Wear Monitor in Smart Manufacturing

**DOI:** 10.3390/s23031240

**Published:** 2023-01-21

**Authors:** Liang Dong, Chensheng Wang, Guang Yang, Zeyuan Huang, Zhiyue Zhang, Cen Li

**Affiliations:** 1 School of Modern Post, Beijing University of Posts and Telecommunications, Beijing 100876, China; 2 School of Artificial and Intelligence, Beijing University of Posts and Telecommunications, Beijing 100876, China; 3 Teaching Affairs Office, Beijing University of Posts and Telecommunications, Beijing 100876, China

**Keywords:** tool wear monitor, multiple sensors, ResNet, channel attention

## Abstract

Tool wear is a key factor in the machining process, which affects the tool life and quality of the machined work piece. Therefore, it is crucial to monitor and diagnose the tool condition. An improved CaAt-ResNet-1d model for multi-sensor tool wear diagnosis was proposed. The ResNet18 structure based on a one-dimensional convolutional neural network is adopted to make the basic model architecture. The one-dimensional convolutional neural network is more suitable for feature extraction of time series data. Add the channel attention mechanism of CaAt1 to the residual network block and the channel attention mechanism of CaAt5 automatically learns the features of different channels. The proposed method is validated on the PHM2010 dataset. Validation results show that CaAt-ResNet-1d can reach 89.27% accuracy, improving by about 7% compared to Gated-Transformer and 3% compared to Resnet18. The experimental results demonstrate the capacity and effectiveness of the proposed method for tool wear monitor.

## 1. Introduction

With the development of the manufacturing industry, machining processes play an increasingly paramount role in the modern manufacturing industry. A major problem of processing is tool wear, which will lead to poor quality of machined parts and low production efficiency [1,2,3,4]. Studies have revealed that 20% of machine downtime can be attributed to different forms of tool wear [5,6]. Specifically, if the tool wear exceeds the failure standard and the tool cannot be superseded in time, it will directly affect the surface quality of the workpiece [7,8]. However, if the tool is replaced too early, it will cause waste and reduce productivity [9]. Tool wear can lead to unexpected downtime and extra costs [10]. Specifically, reliable and accurate tool wear estimates can reduce downtime costs by 10–40% [11]. Therefore, tool wear monitoring is of great significance.

Generally, tool wear diagnostic approaches are differentiated into direct method and indirect method. Results of the direct method are accurate and intuitive, but the operation of the machine tool must be stopped during monitoring, which will prolong the processing time and reduce the production efficiency [12,13]. The monitoring results are easily interfered with by cutting fluid and chips, so the direct method is not suitable for the machine tool processing site [12,13]. The indirect method collects sensor signals, such as force [14,15], vibration [16,17] and acoustic emission [13], extracts data features and establishes a feature map relationship between monitoring signals and tool wear condition.

Generally speaking, feature extraction is mainly divided into two categories: model-based and data-based methods. The model-based method uses a mathematical model to simulate the relationship between tool wear and the machining process to obtain relevant features [18]. Mathematical models are machine learning algorithms, such as the Hidden Markov Model [19], Wiener and Gamma processes [20] and Kalman filtering [21]. Although the model-based approach is successful, it requires a wealth of expertise. This method is susceptible to prior knowledge [22] and limits the maximum utilization of sensors signals.

To overcome these difficulties, data-based approaches have been proposed as more attractive alternatives. The data-based approach has two main advantages. First, it does not require extensive prior knowledge. Second, monitoring sensors can conveniently collect real-time data of cutting tools [23]. Deep Learning (DL) is the epitome of this approach.

In industrial processing, enormous amounts of data are obtained through sensors for tool conditions. DL technology has powerful characteristics, such as powerful nonlinear learning ability. Xu et al. [24] realized multi-scale feature fusion by using the developed parallel convolutional neural network and the channel attention mechanism of the remaining connections considered the weights of different feature graphs. Liu et al. [25] proposed a new neural network model based on Transformer, based on the Transformer model, self-attention mechanism and LSTM. Yin et al. [26] proposed a one-dimensional convolutional neural networks (1D-CNN) and deep Generalized canonical correlation Analysis (DGCCA) for multi-sensor-based tool wear diagnosis. Zhou et al. [27] proposed an improved multi-scale edge marker map neural network, in order to improve the recognition accuracy of TCM medicines based on DL in small samples. Marei et al. [28] proposed a hybrid CNN-LSTM with transfer learning in cutting tool prognostics. Cai et al. [29] proposed a hybrid information system based on Long and Short Term Memory Network (LSTM) for tool wear prediction. Dong et al. [30] proposed a new monitoring method of woodworking tool wear condition by using the limit arithmetic mean filtering method and particle swarm optimization (PSO) backpropagation (BP) neural network algorithm. Phani et al. [31] proposed the deep CNN architecture by selecting appropriate hyperparameters and established the CNN model of tool wear classification by selecting appropriate training parameters. Achyuth et al. [32] proposed support vector machine (SVM) and convolutional neural network (CNN) to analyze the audible signals generated during the machining process to predict the changes of tool wear and workpiece hardness. Shi et al. [33] proposed a new framework for the fusion of multiple heap toxic sparse autoencoders, which mainly consists of a training model and a feature fusion structure. Jian et al. [34] adopted short-time Fourier transform (STFT) for data preprocessing and based on ResNet’s feature layer by layer dimension reduction optimization model.

In this paper, we propose a novel ResNet-based one-dimensional network (CaAt-ResNet-1d) for tool wear condition monitoring. It consists of two kinds of channel attention mechanisms. The dataset reported in the 2010 PHM Data Challenge (PHM Society Conference Data Challenge, provided at https://www.phmsociety.org/competition/phm/10 (accessed on 1 January 2022)) is trained and tested after downsampling. The main contributions of this paper include the following:

1. CaAt-ResNet-1d is realized via ResNet18 of one-dimensional convolutional neural network (1D CNN) and channel attention. Depending on the timing characteristics of tool wear data, ResNet18 is composed of 1D CNN. ResNet residual connections retain the depth advantage of multiple networks and the advantage of shallow networks to avoid degradation problems. In view of the multi-channel features of time series data, the channel attention was in addition to 1D CNN ResNet18 to improve the model’s ability to automatically learn different channel features.
2. The original PHM2010 dataset downsamples and redivides. Three groups of different models were trained and tested on the newly divided dataset, which proved the accuracy and stability of the proposed model.

The rest of the paper is organized as follows: the basic structure of CNN, the residual unit and channel attention are introduced and described in Section 2; Section 3 presents a novel resnet-based one-dimensional network (CaAt-ResNet-1d) for tool wear condition monitoring. In more detail, channel attention mechanisms combining with the residual connections are developed to achieve tool wear classification. Section 4 presents the experimental setup, results and a discussion. Finally, Section 5 presents the conclusion and future work directions.

## 2. Review of Related Work

### 2.1. Convolutional Neural Network (CNN)

CNN occupies a prominent position in the field of computer vision and can also be used in time series. CNN has three basic parts: convolution layer (extraction of image local features), pooling layer (data dimension reduction) and full connection layer (output results).

The convolution operation can be written as:

(1) $$x_{j}^{l}=\sum_{k\in M_{j}}x_{k}^{l-1}*\omega_{kj}^{l}+b_{j}^{l}$$

where $x_{j}^{l}$ represents the *j*th feature map; $x_{k}^{l-1}$ is the *k*th output feature map; $\omega_{kj}^{l}$ means the convolution operation; $M_{j}$ is the input feature size, and $b_{j}^{l}$ is the bias; *l* represents the layer.

The pooling layer of 1D CNN can be written as:

(2) $$x_{j}^{l}=\beta_{j}^{l}*downsampling\left(x_{j}^{l-1}+b_{j}^{l}\right)$$

where $x_{j}^{l}$, $x_{j}^{l-1}$ represent the *j*th feature map of layer *l* and l−1; $\beta_{j}^{l}$ and $b_{j}^{l}$ are the coefficient and additive biases, respectively.

Pooling layer mainly includes several pooling operations: maximum pooling, average pooling and so on. The maximum pooling function of the 1D CNN can be written as:

(3) $$p_{j}=\max_{x_{j}^{l}\in S}\;x_{j}^{l}$$

where $p_{j}^{l}$ represents the value of the pooling operation; *S* represents the width of the pooling layer; $x_{j}^{l}$ represents the input feature map.

The fully connected layer can be written as:

(4) $$z^{lj}=f\left(\sum_{i=1}^{n}W_{ij}^{l-1}a^{\left(l-1\right)i}+b_{j}^{l-1}\right)$$

where $W_{ij}^{l-1}$ is the weight of the kernel; $a^{\left(l-1\right)i}$ is the input from the layer l−1; $b_{j}^{l-1}$ is bias value; f(.) is the activation function.

### 2.2. Residual Unit Connection

**Figure 1:**
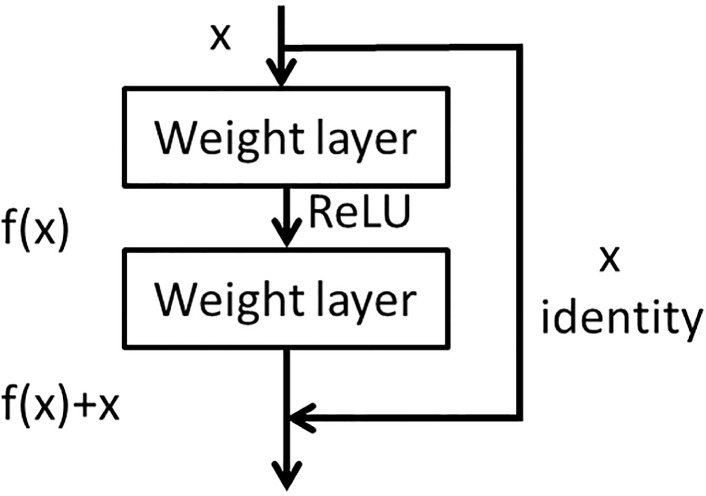
The architecture of residual network block.

The residual unit network [35] adds the result of multiple convolution of the input to the input, as shown in Figure 1. For a multi-layer convolution structure, when the input is *x*, the original feature learned is denoted as H(x)=f(x)+x and one hopes to learn the feature residual f(x)=H(x)−x. When the residual f(x) is 0, the deep network is an identity map H(x)=x. The residual connection not only retains the depth advantage of a deep network, but also retains the advantage of a shallow network to avoid the degradation problem.

### 2.3. Channel Attention

In the DL network, the channel attention mechanism is to focus and learn the importance of different features between channels. Therefore, numerous technologies for channel attention have emerged, such as SENET [36] and CBAM [37].

In SENET, firstly, the feature elements in each channel are globally average pooled to obtain a one-dimensional vector. Secondly, a weight value is obtained through the two convolution layers. The original feature elements of each channel are multiplied by the weight of the corresponding channel to obtain the new feature map. The calculation process can be written as follows:

(5) $$z_{c}=F_{s}q\left(u_{c}\right)=\frac{1}{H}\sum_{i=1}^{H}u_{c}\left(i\right)\;$$

(6) $$s=F_{e}x\left(z,W\right)=\sigma \left(W_{2}\delta \left(W_{1}z\right)\right)\;where,\;W_{1},W_{2}\in R^{\frac{C}{r}\times C}\;$$

where $W_{1}$ represents the full connection layer parameter responsible for compression; $W_{2}$ represents the full connection layer parameter responsible for dimension restoration; σ represents the sigmoid function; δ represents the ReLU function; and the variable *r* is a compression parameter; *C* represents channel; *H* represents the quantity of feature elements.

The channel attention in CBAM differs from SENET. When the channel dimension of the input feature graph is compressed, not only is average pooling considered, but also maximum pooling is introduced as a supplement. Two one-dimensional vectors are calculated via two pooling functions. The one-dimensional vectors are added by the two convolution eigenvectors, respectively, to produce a new eigenvector. Global average pooling has feedback for every pixel on the feature map, while global maximum pooling has feedback of the gradient where the response is the largest in the feature map. The calculation process can be written as:

(7) $$\begin{array}{ll} M_{c}\left(F\right) & =\sigma (\mathrm{CNN}(\mathrm{AvgPool}(F)))+\mathrm{CNN}(\mathrm{MaxPool}(F))\\ & =\sigma \left(W_{1}\left(W_{0}\left(F_{avg}^{c}\right)\right)\right)+W_{1}\left(W_{0}\left(F_{\max}^{c}\right)\right) \end{array}$$

where σ represents the sigmoid function; $W_{0}$, $W_{1}$ represent the CNN weights; *F* represents the input feature map; $F_{\mathrm{avg}}$ and $F_{\max}$ represent the features calculated by global average pooling and global max pooling, respectively.

## 3. The Proposed Method

**Figure 2:**
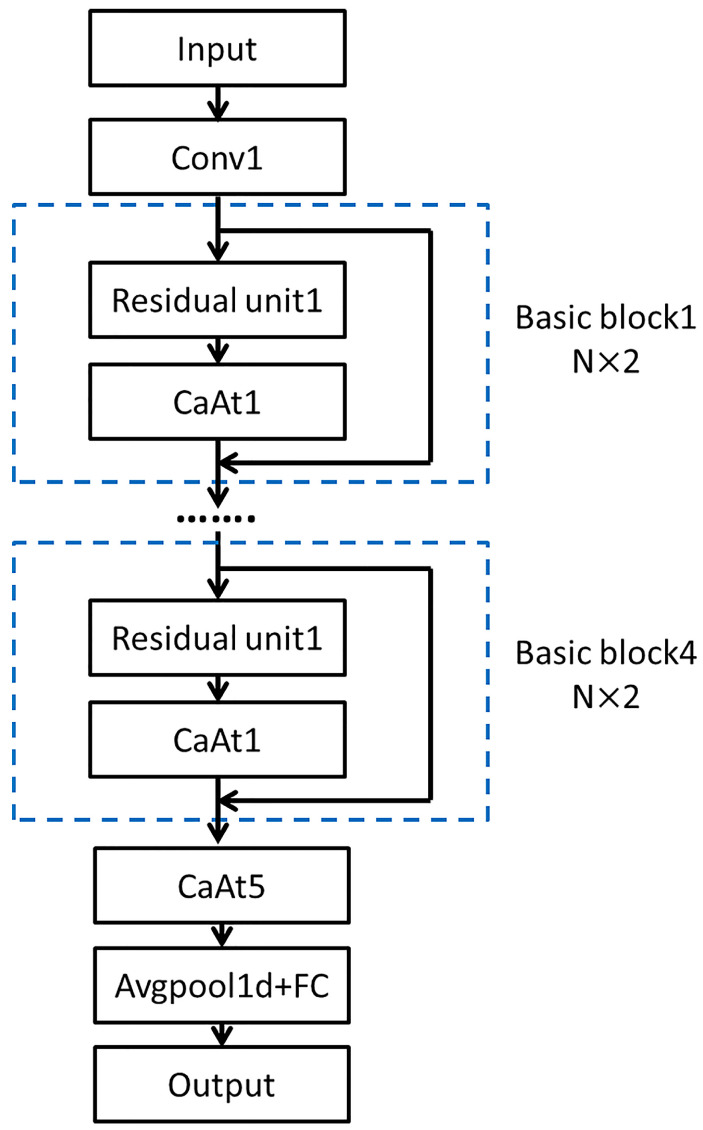
The architecture of the proposed method.

CaAt-ResNet-1d is a modification based on the ResNet18 model. Depending on the characteristics of the tool wear data, the model is composed of 1D CNN and the channel attention mechanism. The channel attention mechanism is specially introduced into the model to extract channel features and finally the full connection layer outputs the evaluation results. The model uses the Adam optimizer, ReLU activation function. The overall diagram is shown in Figure 2.

### 3.1. ResNet with 1D CNN

**Figure 3:**
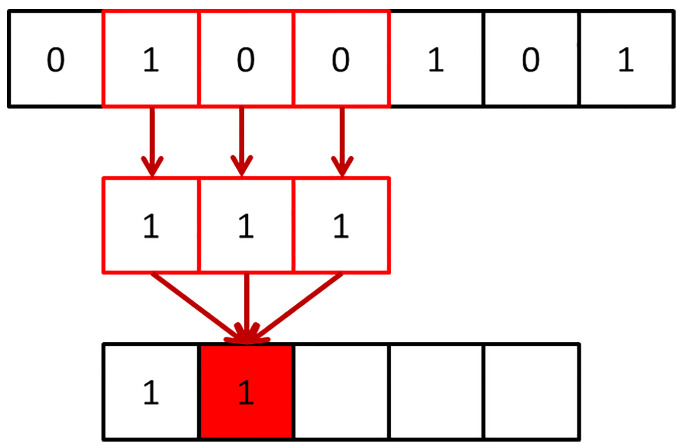
Schematic diagram of 1D CNN.

Concerning one-dimensional CNN, as the name implies, the convolution kernel is one-dimensional. The convolution kernel size of 1D CNN has the exact same dimension as the input data size. The calculation principle of the convolution operation is the same as that of 2D CNN. The difference is that the convolution kernel only has one moving direction. According to the intuitive analysis from the sliding window, the one-dimensional convolution kernel slides along the data length direction, extracting signal features quickly and accurately, as shown in Figure 3.

One-dimensional CNN is more suitable for sequence analysis of signal data. One-dimensional CNN is more apposite than 2D CNN with respect to extracting signal features for tool wear diagnosis. Based on the above reasons, ResNet18, which is widely used and composed of 2D CNN, was adapted to 1D CNN in this paper, as shown in Figure 1.

### 3.2. ResNet with Channel Attention

**Figure 4:**
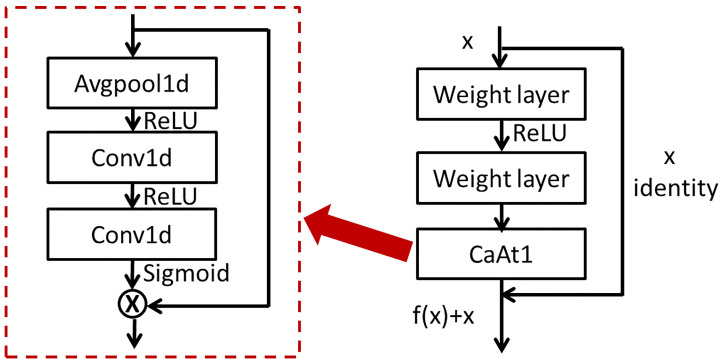
(**left**) The architecture of CaAt1. (**right**) The architecture of Basic block.

The Basic block consists of a residual block and a channel attention mechanism, as shown in Figure 4. The residual block and channel attention structure in each Basic block are the same, respectively, with different parameters. CaAt1 firstly performs global average pooling on each channel to obtain feature vectors. Secondly, the weight value of the feature vector obtained by the two convolution layers is multiplied by the original feature vector to obtain a new feature vector. The local depth and channel depth feature are extracted by using the advantage of residual connection.

**Figure 5:**
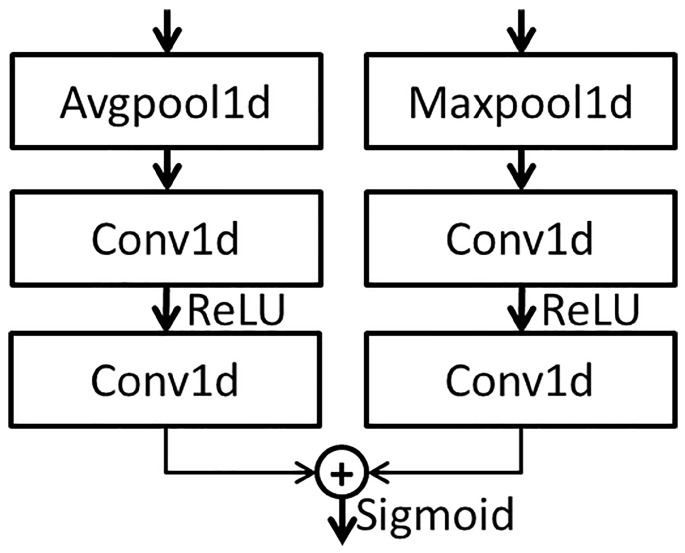
The architecture of CaAt5.

The features extracted from the multi-layer residual network block are input into the CaAt5 structure. The structure diagram of CaAt5 is illustrated in Figure 5. When extracting channel features, not only is average pooling considered, but also maximum pooling is introduced as a supplement. The feature vectors are generated by two pooling functions, respectively, and the new feature vectors are generated by adding the feature vectors after two convolution calculations. Global average pooling has feedback for every pixel on the feature map, while global maximum pooling has feedback of the gradient where the response is the largest in the feature map. Feature calculation is carried out for each pixel on the multi-channel and the one with the largest response in the feature map. This calculation method is more suitable for the adaptive feature extraction of multi-channel time sequence data and automatically learns the importance of features between different channels, as shown in the overall figure in Figure 1.

## 4. Experiments and Results

### 4.1. Dataset Description

**Figure 6:**
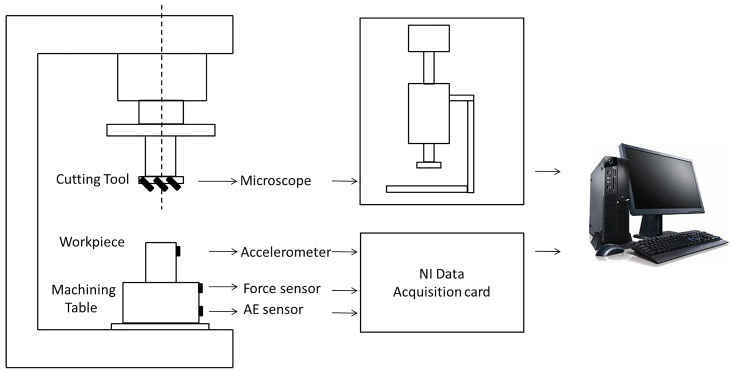
Schematic diagram of experimental setup and the data collected system.

The dataset which analyzes the performance of the proposed method is originally made available from the PHM2010 data challenge. It is obtained from a high-speed CNC machine which uses a three slot ball head tungsten carbide tool on the surface of stainless steel workpiece processing. During milling processing, the three-way dynamometer is installed between the workpiece and the processing table to measure the cutting force in X, Y and Z directions, the piezoelectric acceleration sensor is installed on the workpiece to collect vibration signals in X, Y and Z directions, the Kistler acoustic emission sensor is installed on the processing table to monitor and record high-frequency stress wave changes, as shown in Figure 6.

The details of CNC equipment are shown in Table 1, the setting of milling conditions is shown in Table 2 and the signal data collected by the corresponding sensors of different channels are shown in Table 3.

The workpiece machining surface used in the milling process is a square with a length of 108 mm and the distance of each milling of 108 mm is marked as one cutting. After each cutting, the tool wear amount of the back cutting surface is measured with a microscope and recorded as the wear result. The number for the cutting time in each process is 315.

**Figure 7:**
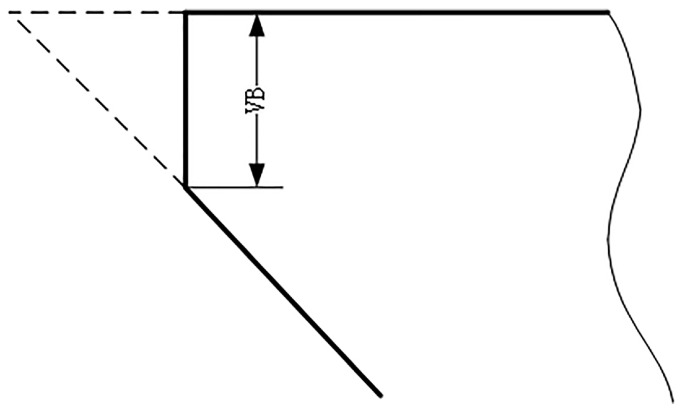
Schematic drawing of blunt standard.

**Figure 8:**
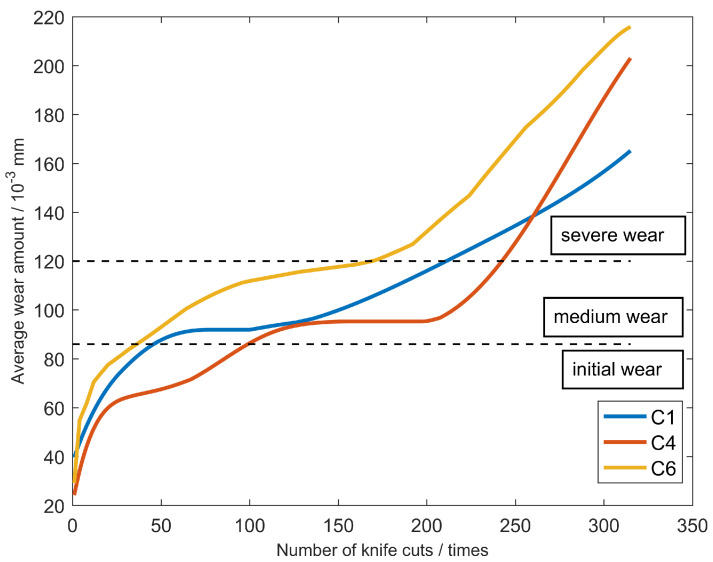
The measured tool wear of the three milling cutters.

A total of six ball-end carbide milling cutters are recorded as C1, C2, C3, C4, C5 and C6. Only C1, C4 and C6 are used in the model evaluation. The three-way force signal, three-way vibration signal and acoustic emission signal of the tool are, respectively, collected in each process, as shown in Table 3. Considering the convenience of measurement in industrial application, the wear width VB of 1/2 cutting amount on the back tool face is selected as the standard parameter of tool blunting, as shown in Figure 7. For carbide cutting tools, VB above 0.3 mm reaches the standard blunt and the tool must be replaced. The average wear value of the rear tool was calculated as the wear label. Maximum wear of the initial wear stage is 0.086 mm. When the wear amount is greater than 0.12 mm, the tool has reached the stage of severe wear and must be replaced immediately. The middle wear stage is between 0.086 mm and 0.12 mm.The tool wears curves of C1, C4 and C6 are shown in Figure 8.

As shown in Table 1, spindle speed is 10,400 RPM, indicating that the tool rotates 173 revolutions per second. The data sampling rate was 50 khz; consequently, approximately 289 data points are collected per rotation. The data points are too redundant and the computation time is added without value. Therefore, it is indispensable to re-select the appropriate signal length, which should be as brief as possible and be able to express all the captured features. After calculation, the most appropriate length of the signal data points is 256. The data of each milling process are analysed and calculated, respectively. Taking C1 as an example, the number of cutting events in the initial wear stage is approximately 50, the number of the middle wear stage is about 160 and the number of the severe wear stage is close to 100. If training data are generated by randomly sampling from the complete dataset, the number of initial wear categories would be approximately 16%, about 51% of the samples would be in the middle wear category and about 33% would be in the severe wear category. The training sample generates the problem of class data imbalance. To solve this problem, the dataset needs to be redefined using appropriate downsampling operations.

Because a sufficient amount of data are collected in each milling process, downsampling operations of the three categories can be performed separately. As an example, more than 200,000 data points are collected per walk in C1 and 50 runs were in the initial wear stage. Thus, a total of 10 million data points are gathered during the initial wear stage. In order to ensure category balance, the initial wear stage is downsampled at a rate of 8% and about 3000 samples of the initial wear stage are obtained. With the same downsampling strategy, the downsampling rates of 2% and 4% are adopted in the middle wear and severe wear stages, respectively. The C1 dataset redefined a dataset consisting of 8954 samples, including 2990 samples in the initial wear stage, 3006 samples in the middle wear stage and 2958 samples in the severe wear stage.

### 4.2. Training and Test

After the dataset is downsampled, the average wear value of each cutting event is calculated for label establishment. Initial wear, middle wear and severe wear correspond to labels 1, 2 and 3, respectively.

Raw data collected from seven channels are worked in the CaAt-ResNet-1d model for multi-sensor tool wear diagnosis. In order to fully verify the experiment, three sets of verification experiments are conducted on C1, C2 and C3 datasets, as shown in Table 4. In the first set of experiments, data from C1 and C4 cutters (a total of 17,684 samples) are used as a training set to generate model M1+4, which is tested using data from C6 cutters. In the second set of experiments, data from C1 and C6 cutters (17,190 samples in total) are invoked as a training set to produce model M1+6, which is tested using data from C4 cutters. In the third experiment, the training set of C4 and C6 tool data (17,414 samples in total) are used to obtain the model M4+6 and the data of the C1 tool are utilized to test the model.

**Figure 9:**
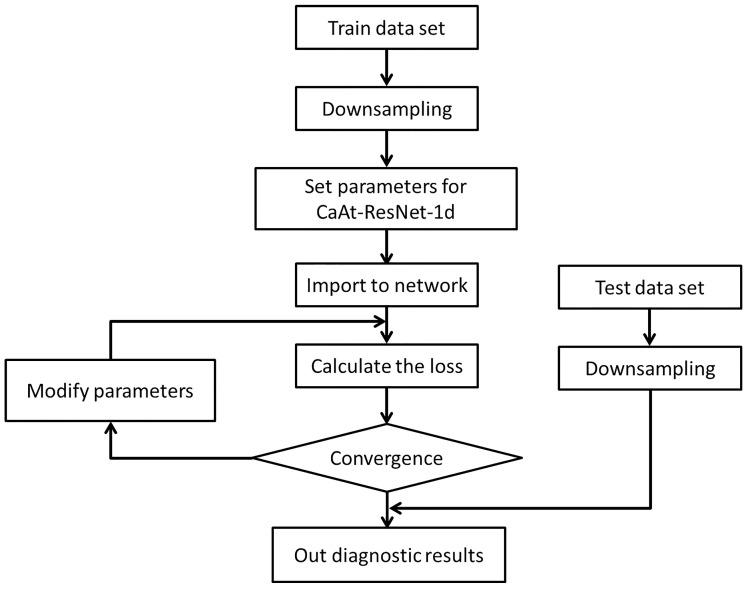
Flowchart of the steps for the training and test.

The computer system is Ubuntu 18.04, which uses two nVidia 1080Ti GPUs for parallel training. The general flow is shown in Figure 9.

1. The collected C1, C4 and C6 datasets are subsampled and divided into new datasets;
2. CaAt-ResNet-1d model initialization parameters, learning rate is 0.0001, detailed parameters are shown in Table 5;
3. After data input to the model, the loss value, reverse transmission and correction of the hyperparameter are calculated;
4. The model and output the evaluation results are tested.

Binary cross entropy is used as a loss function in the evaluation and its mathematical expression can be expressed as follows:

(8) $$l_{n}=-\left(y_{n}*\log\left(\overset{\wedge }{y}\;\right)+\left(1-y_{n}\right)*\log\left(1-{\overset{\wedge }{y}\;}_{n}\right)\right)$$

(9) $$\mathrm{loss}\left(z,y\right)=\mathrm{mean}\left\{l_{0},l_{1},l_{2}\ldots ,l_{N-1}\right\}$$

Accuracy is used as the evaluation index of the experiment and the test results can be extensively quantified by accuracy. The specific formula is shown as follows:

(10) $$A_{CC}=\frac{\sum TP}{TP+TN+FP+FN}$$

where $A_{CC}$ represents the overall accuracy of the evaluation. TP, TN, FP and TN represent the number of true positives, false negatives, false positives and true negatives

### 4.3. Experiment Results

In this part, we construct a CaAt-ResNet-1d model and evaluate the tool condition through the actual tool data. C1, C4 and C6 in three test experiments (M4+6, M1+6 and M1+4) are fully trained and tested, respectively. In order to further assess the performance of CaAt-ResNet-1d, comparison is made with other popular algorithms, LSTM [38], GRU [39], Gated-Transformer [40] and Resnet18 [35]. Table 6 presents the results of different algorithms, respectively.

The experimental results demonstrate that different algorithm models produce different accuracy. The accuracy of the CaAt-ResNet-1d algorithm is greater than of other popular algorithms in the three experiments, respectively. In the M1+4 tested dataset, the proposed method improves by about 4% compared to LSTM, 4% compared to GRU, 12% compared to Gated-Transformer and 5% compared to Resnet18. In the M1+6 tested dataset, the proposed method improves by about 13% compared to LSTM, 9% compared to GRU, 20% compared to Gated-Transformer and 3% compared to Resnet18. In the M4+6 tested dataset, the proposed method improves by about 7% compared to LSTM, 2% compared to GRU, 6% compared to Gated-Transformer and 3% compared to Resnet18. The highest accuracy of the LSTM model is 81.52%, GRU model is 85.82%, Gated Transformer model is 81.95% and ResNet18 model is 85.12%. The highest accuracy of the CaAt-ResNet-1d model is 89.27%, which is more effective than other algorithms.

### 4.4. Discussion

In this study, an improved CaAt-ResNet-1d model is proposed for the PHM2010 dataset. We completed the experimental verification on three sets of downsampling datasets. The CaAt-ResNet-1d algorithm behaves more efficiently than LSTM, GRU, Gated-Transformer and Resnet18. The accuracy of Gated-Transformer is the worst; a possible reason is that the dataset sample is not sufficient. Gated-Transformer with Transformer [41] as the core requires a large number of samples to reflect the superiority of the algorithm, so it is not suitable for the dataset of small samples like PHM2010. LSTM and GRU are suitable for time series data monitoring, but their accuracies are not the highest. A possible reason is that the algorithm is too simple and does not extract rich features. Compared to the baseline Resnet18, CaAt-ResNet-1d has been improved to obtain the highest accuracy. The reasons for this may be as follows: 1. Residual connections retain the depth advantage of multiple networks and the advantage of shallow networks to avoid degradation problems. 2. One-dimensional CNN and channel attention are more suitable for feature extraction of multi-sensor series data. There are differences in the accuracy of CaAt-ResNet-1d on the results of M1+4, M1+6, M4+6 datasets that cannot be ignored. LSTM, GRU, Gated-Transformer and Resnet18 also have accuracy gaps that cannot be ignored in the three test datasets, as shown in Table 7. The reasons for this may be as follows: the experiment datasets could not fully include various feature spaces. In addition, it is worth noting that the current results do not meet real-time machining requirements. The reasons for this may be as follows: 1. The signal data collected by the sensor is vulnerable to the influence of the milling process, resulting in the destruction of the actual characteristics of the data. 2. The dataset does not collect enough rich data samples and could not fully include various feature spaces, resulting in failing to meet the standard for real-time detection. On the algorithm level, we can try to design a more reasonable network to achieve the purpose of real-time detection. For example, the combination design of CaAt-ResNet-1d and GRU may obtain more ideal accuracy.

## 5. Conclusions and Future Work

A new tool wear diagnosis method based on multi-sensor data of CaAt-ResNet-1d is proposed. Multiple sensors are utilized to collect the original data of the machine and provide rich feature information. Features are extracted adaptively through 1D CNN and channel attention mechanism and deep data features are extracted through residual network blocks to output evaluation results. By training and testing on the PHM2010 dataset, the proposed method achieves 89.27% accuracy, indicating that this method has an improved ability in accuracy. The following conclusions can be obtained:

1. Based on the data of multiple sensor signals (such as sound, vibration and force), features are extracted adaptively through 1D CNN without any prior knowledge. The channel attention mechanism is added to the network model to extract features between different channels. The residual network block can extract the deep features of the data.
2. The original data were downsampled and re-divided to maintain the balance of data categories. The training and verification of the model were completed under three sets of different datasets, respectively, proving the superiority of the proposed algorithm.

This study can be used for tool wear applications on various machining studies, but the algorithm’s limitations remain. As an example, whether there are other methods to improve the accuracy remains to be further explored and whether the identification accuracy can be achieved in the actual application process has not been verified. Future research work includes further exploration of ways to improve accuracy and practical applications.

## Figures and Tables

**Table 1:** Details of the CNC equipment.

Name	Type
CNC machine	Roders Techy RFM760
Cutter	Ball nose tungsten Carbide Cutter
Dynamometer	Kistler 9265B
Vibration	Kistler 8636C
Accelerometer	Kistler 5019
Data Collector	NI DAQ PCI 1200
Wear measuring device	LEICA MZ12 microscope

**Table 2:** Working condition setting of milling process.

Parameter Name	Value
Spindle speed r/min	10,400
Rate of feed mm/min	1555
Depth of cutting mm	0.2
Width of cutting mm	0.126
Frequency of sampling KHz	50

**Table 3:** Signal channels and corresponding measured data.

Signal Channel	Measured data
Channel 1	Vx—vibration with the x-axis
Channel 2	Vy—vibration with the y-axis
Channel 3	Vz—vibration with the z-axis
Channel 4	Fx—Cutting force with the x-axis
Channel 5	Fy—Cutting force with the y-axis
Channel 6	Fz—Cutting force with the z-axis
Channel 7	AE—acoustic emission

**Table 4:** Strategy for training and testing data.

Model	Training Dataset	Test Data
M1+4	C1 and C4	C6
M1+6	C1 and C6	C4
M4+6	C4 and C6	C1

**Table 5:** The specific parameters of the developed method.

Name		Filters	Kernel Size/Stride	Activation Function
Conv1	Conv1d	64	7/2	ReLU
Basic block1	Conv1d	64	3/1	ReLU
	Conv1d	64	3/1	ReLU
CaAt1	AvgPool1d		1/0	
	Conv1d	4	1/0	ReLU
	Conv1d	64	1/0	Sigmoid
Basic block2	Conv1d	128	3/2	ReLU
	Conv1d	128	3/1	ReLU
CaAt2	AvgPool1d		1/0	
	Conv1d	8	1/0	ReLU
	Conv1d	128	1/0	Sigmoid
Basic block3	Conv1d	256	3/2	ReLU
	Conv1d	256	3/1	ReLU
CaAt3	AvgPool1d		1/0	
	Conv1d	16	1/0	ReLU
	Conv1d	256	1/0	Sigmoid
Basic block4	Conv1d	512	3/2	ReLU
	Conv1d	512	3/1	ReLU
CaAt4	AvgPool1d		1/0	
	Conv1d	32	1/0	ReLU
	Conv1d	512	1/0	Sigmoid
CaAt5	AvgPool1d		1/0	
	AvgPool1d		1/0	
	Conv1d	8	1/0	ReLU
	Conv1d	512	1/0	Sigmoid
AvgPool1d			1/0	

**Table 6:** Performance of different algorithms for the three cutter model.

Method	$A_{\mathrm{CC}}$ (M1 + 4)	$A_{\mathrm{CC}}$ (M1 + 6)	$A_{\mathrm{CC}}$ (M4 + 6)
LSTM	81.52	76.53	81.24
GRU	81.48	80.41	85.82
Gated-Transformer	72.84	69.51	81.95
Resnet18	80.52	85.92	85.12
CaAt-ResNet-1d	85.25	89.27	87.98

**Table 7:** The maximum accuracy difference of different algorithms.

Method	Gap
LSTM	4.99
GRU	5.41
Gated-Transformer	12.44
Resnet18	5.40
CaAt-ResNet-1d	4.02

## Data Availability

The data presented in this study are available on request from the corresponding author. The data are PHM2010: https://www.phmsociety.org/competition/phm/10 (accessed on 1 January 2022).

## Abbreviations

The following abbreviations are used in this manuscript:

PHM	Prognostics Health Management
DL	Deep Learning
ML	Machine Learning
